# O-05 - WHEN TEST POSITIVITY MISLEADS: USE OF THE GOLDSTEIN INDEX AS A MEASURE OF RESPIRATORY PATHOGEN INTENSITY IN PRIMARY CARE IN SCOTLAND

**DOI:** 10.1093/pubmed/fdag046.005

**Published:** 2026-07-09

**Authors:** Dr Josie Evans, Dr Ronan McCabe, Dr Kimberly Marsh

**Affiliations:** Public Health Scotland; Public Health Scotland; Public Health Scotland

## Abstract

**Background:**

Since October 2022, the nationally-representative CARI sentinel surveillance system in >100 GP practices in Scotland has used test-positivity (TP) to monitor 10 pathogens among patients presenting with acute respiratory infection (ARI). TP reflects relative pathogen prevalence among those tested but does not account for substantial variation in background ARI prevalence. The Goldstein index (GI), which combines TP with population-based ARI rates, may be a more valid measure of pathogen intensity.

**Objectives:**

To compare GI and TP for selected infectious respiratory pathogens in Scotland.

**Methods:**

GI, calculated as the product of CARI TP and Scotland-level ARI rates, was compared with TP for selected seasonal and non-seasonal pathogens. ARI rates are derived from GP consultation rates and are well-validated.

**Results:**

COVID-19 showed a distinct temporal pattern compared with typical seasonal respiratory pathogens, with activity peaking during summer months when background ARI rates were low. Consequently, high TP did not translate into high population burden. During summer COVID-19 peaks, GI values were moderate: in July 2024, TP reached 22.3% (95%CI:19.0–26.0) with a GI of 28.8/100,000, while in August 2025 TP reached 20.5% (95%CI:15.9–25.9) with a GI of 20.0/100,000.

In contrast, winter-peaking respiratory pathogens exhibited lower TP but higher GI, due to higher ARI rates. Seasonal coronavirus TP peaked at 14.2% (95%CI:12.1-16.7) in February 2024, but reached GI of 34.4/100,000, substantially higher than summer COVID-19 GI. Influenza showed a similar winter pattern, with peaks in TP coinciding with high ARI rates and consequently high GI.

**Conclusion:**

GI is a more meaningful measure of pathogen intensity than TP, conveying population disease burden rather than relative prevalence among those tested. For non-seasonal pathogens such as COVID-19, TP can overstate intensity during low-ARI periods and obscure meaningful seasonal differences in impact. Incorporating GI into routine surveillance supports clearer risk communication and improved public health decision-making.

